# Chronic Diseases and Conditions Related to Alcohol Use

**DOI:** 10.35946/arcr.v35.2.06

**Published:** 2014

**Authors:** Kevin D. Shield, Charles Parry, Jürgen Rehm

**Affiliations:** **Kevin D. Shield, M.H.Sc.,***is a Ph.D. student in medical science at the University of Toronto. He also is affiliated with the Centre for Addiction and Mental Health (CAMH) in Toronto, Canada.*; **Charles Parry, Ph.D.,***is the director of the Alcohol & Drug Abuse Research Unit at the South African Medical Research Council, Cape Town, South Africa, and extraordinary professor in the Department of Psychiatry, Stellenbosch University, Cape Town, South Africa.*; **Jürgen Rehm, Ph.D.,***is director of the Social and Epidemiological Research Department at the Centre for Addiction and Mental Health, chair and professor in the Dalla Lana School of Public Health, University of Toronto, Canada, and section head at the Institute for Clinical Psychology and Psychotherapy, Technische Universität Dresden, Dresden, Germany.*

**Keywords:** Alcohol consumption, alcohol use frequency, chronic diseases, disorders, mortality, morbidity, alcohol-attributable fractions (AAF), risk factors, relative risk, AOD-induced risk, cancers, neuropsychiatric disorders, cardiovascular diseases, digestive diseases, diabetes, ischemic stroke, ischemic heart disease, burden of disease

## Abstract

Alcohol consumption is a risk factor for many chronic diseases and conditions. The average volume of alcohol consumed, consumption patterns, and quality of the alcoholic beverages consumed likely have a causal impact on the mortality and morbidity related to chronic diseases and conditions. Twenty-five chronic disease and condition codes in the International Classification of Disease (ICD)-10 are entirely attributable to alcohol, and alcohol plays a component-risk role in certain cancers, other tumors, neuropsychiatric conditions, and numerous cardiovascular and digestive diseases. Furthermore, alcohol has both beneficial and detrimental impacts on diabetes, ischemic stroke, and ischemic heart disease, depending on the overall volume of alcohol consumed, and, in the case of ischemic diseases, consumption patterns. However, limitations exist to the methods used to calculate the relative risks and alcohol-attributable fractions. Furthermore, new studies and confounders may lead to additional diseases being causally linked to alcohol consumption, or may disprove the relationship between alcohol consumption and certain diseases that currently are considered to be causally linked. These limitations do not affect the conclusion that alcohol consumption significantly contributes to the burden of chronic diseases and conditions globally, and that this burden should be a target for intervention.

Alcohol has been a part of human culture for all of recorded history, with almost all societies in which alcohol is consumed experiencing net health and social problems (McGovern 2009; Tramacere et al. 2012*b*, *c*). With the industrialization of alcohol production and the globalization of its marketing and promotion, alcohol consumption and its related harms have increased worldwide (see Alcohol Consumption Trends, in this issue). This has prompted the World Health Organization (WHO) to pass multiple resolutions to address this issue over the past few years, including the World Health Assembly’s Global Strategy to Reduce the Harmful Use of Alcohol, which was passed in May 2010. Of growing concern are noncommunicable chronic diseases and conditions that have been shown to contribute substantially to the alcohol-attributable burden of disease (Rehm et al. 2009). Specifically, in 2004 an estimated 35 million deaths and 603 million disability-adjusted life-years (DALYs) lost were caused by chronic diseases and conditions globally (WHO 2008); alcohol was responsible for 3.4 percent of the deaths and 2.4 percent of DALYs caused by these conditions (Parry et al. 2011). To address the burden of chronic diseases and conditions, the United Nation (UN) General Assembly passed Resolution 64/265 in May of 2010, calling for their prevention and control (UN 2010). This resolution is intended to garner multisectoral commitment and facilitate action on a global scale to address the fact that alcohol (together with tobacco, lack of exercise, and diet) plays a significant role in chronic diseases and conditions. It is noteworthy that cardiovascular diseases, cancers, and diabetes in particular have been highlighted for targeted action (UN 2010) because alcohol is a risk factor for many cardiovascular diseases and cancers and has both beneficial and detrimental effects on diabetes and ischemic cardiovascular diseases,^1^ depending on the amount of alcohol consumed and the patterns of consumption.

Building on previous reviews concerning alcohol and disease (Rehm et al. 2003*a*, 2009), this article presents an up-to-date and in-depth overview of the relationship of alcohol consumption and high-risk drinking patterns and the initiation/exacerbation and treatment of various chronic diseases and conditions. It also assesses the methods used to calculate the impact of alcohol consumption on chronic diseases and conditions.

## Alcohol Consumption As a Risk Factor for Chronic Diseases and Conditions

**Figure 1:**
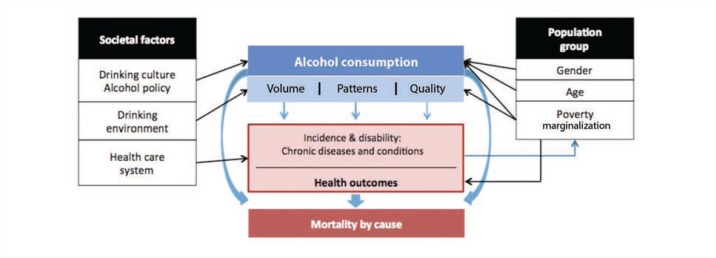
Causal model of alcohol consumption, intermediate mechanisms, and long-term consequences, as well as of the influence of societal and demographic factors on alcohol consumption and alcohol-related harms resulting in chronic diseases and conditions. SOURCE: Adapted from Rehm et al. 2010*a*.

Figure 1 presents a conceptual model of the effects of alcohol consumption on morbidity and mortality and of the influence of both societal and demographic factors on alcohol consumption and alcohol-related harms resulting in chronic diseases and conditions (adapted from Rehm et al. 2010*a*). According to this model, two separate, but related, measures of alcohol consumption are responsible for most of the causal impact of alcohol on the burden of chronic diseases and conditions—overall volume of alcohol consumption and patterns of drinking. The overall volume of alcohol consumption plays a role in all alcohol-related diseases, whereas drinking patterns only affect ischemic cardiovascular diseases. In addition to the overall volume and pattern of consumption, the quality of the alcoholic beverages consumed also may influence mortality and morbidity from chronic diseases and conditions. However, this pathway is of less importance from a public health perspective (Lachenmeier and Rehm 2009; Lachenmeier et al. 2007) because it has a much smaller impact than the other two factors.

The effects of overall volume of alcohol consumed, consumption patterns, and quality of the alcoholic beverages consumed on mortality and morbidity from chronic diseases and conditions are mediated by three main mechanisms.

These include the following:

- The toxic and beneficial biochemical effects of beverage alcohol (i.e., ethanol) and other compounds found in alcoholic beverages;
- The consequences of intoxication; and
- The consequences of alcohol dependence.

These intermediate mechanisms have been reviewed in more detail by Rehm and colleagues (2003*a*).

## Chronic Diseases and Conditions Related to Alcohol

### Chronic Diseases and Conditions Entirely Attributable to Alcohol

Of the chronic diseases and conditions causally linked with alcohol consumption, many categories have names indicating that alcohol is a necessary cause—that is, that these particular diseases and conditions are 100 percent alcohol attributable. Of these, alcohol use disorders (AUDs)—that is, alcohol dependence and the harmful use of alcohol as defined by the *International Classification of Disease, Tenth Edition* (ICD–10)—certainly are the most important categories, but many other diseases and conditions also are entirely attributable to alcohol (see table 1).

### Chronic Diseases and Conditions for Which Alcohol Is a Component Cause

Alcohol is a component cause for more than 200 other diseases and conditions with ICD–10 three-digit codes—that is, alcohol consumption is not necessary for the diseases to develop (Rehm et al. 2010*a*). For these conditions, alcohol shows a dose-response relationship, where the risk of onset of or death from the disease or condition depends on the total volume of alcohol consumed (Rehm et al. 2003*a*). Table 2 outlines these chronic diseases and conditions that are associated with alcohol consumption and lists the source of the relative risk (RR) functions if the chronic disease or condition is included as an alcohol-attributable harm in the 2005 Global Burden of Disease (GBD) Study.^2^ Several of these chronic diseases and conditions are singled out for further discussion in the following sections to highlight alcohol’s causative or protective role.

## Specific Chronic Diseases and Conditions Associated With Alcohol Consumption

### Malignant Neoplasms

**Figure 2:**
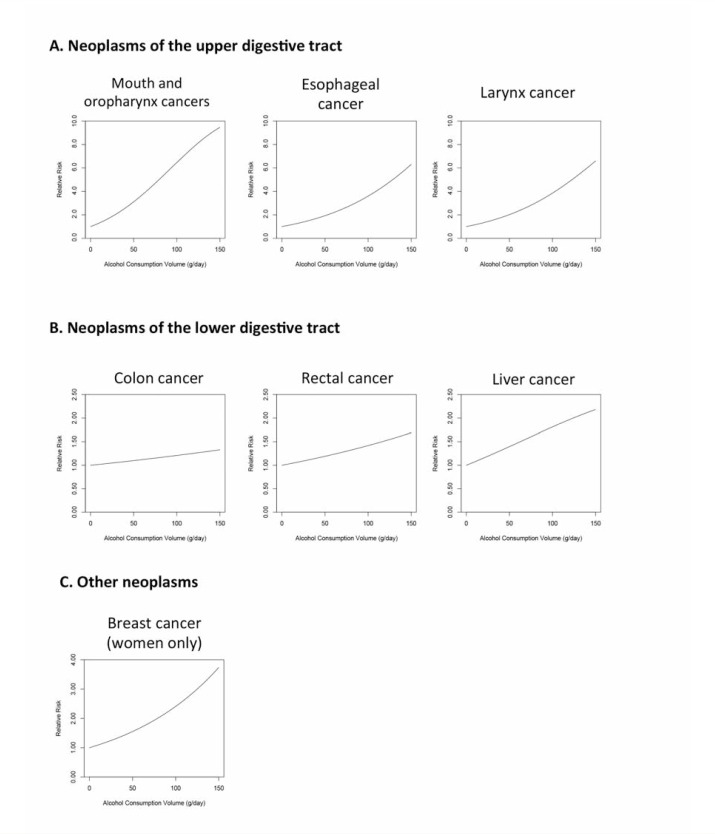
The relationship between increasing amounts of average daily alcohol consumption and the relative risk for cancer, with lifetime abstainers serving as the reference group. SOURCE: Lim et al. 2012.

The relationship between alcohol consumption and cancer already was suggested in the early 20th century, when Lamy (1910) observed that patients with cancer either of the esophagus or of the cardiac region were more likely to be alcoholics. The accumulation of evidence supporting the relationship between ethanol and cancers led the International Agency for Research on Cancer (IARC) to recognize the cancer-inducing potential (i.e., carcinogenicity) of ethanol in animal models and to conclude that alcoholic beverages are carcinogenic to humans (IARC 2008). Specifically, the GBD study found that alcohol increased the risk of cancers of the upper digestive track (i.e., mouth and oropharynx, esophagus, and larynx), the lower digestive track (i.e., colon, rectum, and liver), and the female breast (see figure 2). More up-to-date systematic reviews and meta-analyses on alcohol consumption and the risk of developing cancer have been published by Fedirko and colleagues (2011) for colorectal cancer, Islami and colleagues (2011) for esophageal squamous cell carcinoma, Islami and colleagues (2010) for laryngeal cancer, and Tramacere and colleagues (2010) and Turati and colleagues (2010) for oral and pharyngeal cancers.

A recent meta-analysis also has indicated that alcohol consumption is significantly linked to an increased risk of developing prostate cancer in a dose-dependent manner (Rota et al. 2012); this observation is consistent with previous meta-analyses concluding that alcohol consumption and the risk for prostate cancer are significantly correlated (Dennis 2000; Fillmore et al. 2009). Additional research, however, is required on the biological pathways to prove the role of alcohol consumption in the development of this type of cancer.

Evidence also has suggested that stomach cancer may be linked to ethanol consumption (Bagnardi et al. 2001; Tramacere et al. 2012*a*); however, the findings have not been unequivocal. Thus, two recent meta-analyses found no association between alcohol drinking status (i.e., drinkers compared with non-drinkers) and risk of gastric cardia adenocarcinoma (Tramacere et al. 2012*a*, *d*). However, one meta-analysis did find an association between heavy alcohol consumption and the risk of this type of cancer (Tramacere et al. 2012*a*).

For several types of cancer investigators have found a nonsignificant positive association with alcohol consumption, including endometrial (Bagnardi et al. 2001; Rota et al. 2012), ovarian (Bagnardi et al. 2001), and pancreatic cancers (Bagnardi et al. 2001). However, because the relationship at least between alcohol consumption and endometrial and pancreatic cancer is modest (i.e., the point estimates of RR are low, even at high levels of average daily alcohol consumption), additional studies with large numbers of participants are needed to accurately assess the relationship (Bagnardi et al. 2001). The relationship between alcohol consumption and bladder and lung cancers is even less clear, with one meta-analysis finding that alcohol significantly increases the risk for both types of tumors (Bagnardi et al. 2001), whereas more recent meta-analyses have found no significant association between alcohol consumption and the risk of bladder cancer (Pelucchi et al. 2012) or the risk of lung cancer in individuals who had never smoked (Bagnardi et al. 2001). These conflicting results may stem from the studies in the more recent meta-analyses adjusting for smoking status when assessing the risk relationship between alcohol and these cancers within individual observational studies (Bagnardi et al. 2001; Pelucchi et al. 2012).

The biological pathways by which alcohol increases the risk of developing cancer depends on the targeted organ and are not yet fully understood. Factors that seem to play a role include the specific variants of alcohol-metabolizing enzymes (i.e., alcohol dehydrogenase [ADH], aldehyde dehydrogenase [ALDH], and cytochrome P450 2E1) a person carries or the concentrations of estrogen as well as changes in folate metabolism and DNA repair (Boffetta and Hashibe 2006). For example, a deficiency in ALDH2 activity in people carrying a gene variant (i.e., allele) called *ALDH2 Lys487* contributes to an elevated risk of esophageal cancer from alcohol consumption (Brooks et al. 2009). Because the *ALDH2 Lys487* allele is more prevalent in Asian populations (i.e., Japanese, Chinese, and Koreans) (Eng et al. 2007), and ALDH2 is hypothesized to impact the risk associated with alcohol for all cancers, studies should account for the presence of this allele when assessing the risk relationship between alcohol consumption and the development of any form of cancer.

However, it is important to note that alcohol not only increases the risk of cancer but also may lower the risk of certain types of cancer. For example, meta-analyses of observational studies have found that alcohol significantly decreases the risk of renal cell carcinoma (Bellocco et al. 2012; Song et al. 2012), Hodgkin’s lymphoma (Tramacere et al. 2012*c*), and non-Hodgkin’s lymphoma (Tramacere et al. 2012*b*). Alcohol’s protective effect for renal cancer is thought to be mediated by an increase in insulin sensitivity, because light to moderate alcohol consumption has been associated with improved insulin sensitivity (Davies et al. 2002; Facchini et al. 1994; Joosten et al. 2008). Insulin resistance may play a key role in the development of renal cancer because people with diabetes, which is characterized by insulin resistance, have an increased risk of renal cancers (Joh et al. 2011; Lindblad et al. 1999). The mechanisms underlying alcohol’s protective effect on the risk of developing Hodgkin’s lymphoma and non-Hodgkin’s lymphoma currently are unknown (Tramacere et al. 2012*b*, *c*). Thus, these observed protective effects should be interpreted with caution because the underlying biological mechanisms are not understood and confounding factors and/or misclassification of abstainers within observational studies may be responsible for these effects.

### Diabetes

Research has found that moderate alcohol consumption is associated with a reduced risk of type 2 diabetes^3^ (Baliunas et al. 2009). Because development of insulin resistance is key in the pathogenesis of type 2 diabetes, it is thought that moderate alcohol consumption protects against the disorder by increasing insulin sensitivity (Hendriks 2007). Such an alcohol-related increase in insulin sensitivity has been found in observational studies as well as in randomized controlled trials (Davies et al. 2002; Kiechl et al. 1996; Lazarus et al. 1997; Mayer et al. 1993; Sierksma et al. 2004). Alternative explanations for the protective effect of moderate alcohol consumption involve an increase in the levels of alcohol metabolites, such as acetaldehyde and acetate (Sarkola et al. 2002); an increase in high-density lipoprotein (HDL)^4^ (Rimm et al. 1999); and the anti-inflammatory effects of alcohol consumption (Imhof et al. 2001). It is important to note, however, that although there is reason to believe that alcohol consumption is causally linked to reduced risk of type 2 diabetes, it currently is unclear whether alcohol consumption itself is a protective factor or if moderate drinking is a marker for healthy lifestyle choices that may account for some of the observed protective effect.

**Figure 3:**
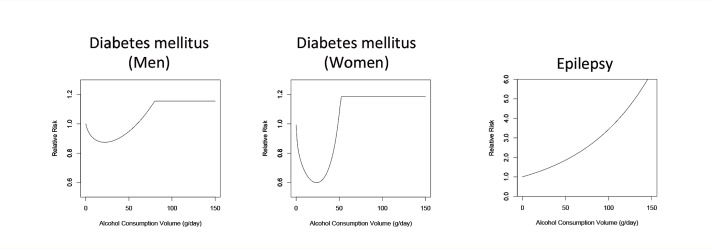
The relationship between increasing amounts of average daily alcohol consumption and the relative risk for diabetes and epilepsy, with lifetime abstainers serving as the reference group. SOURCE: Lim et al. 2012.

Furthermore, the effects of alcohol consumption on risk of diabetes are dose dependent (see figure 3). Thus, in observational studies consumption of large amounts of alcohol has been related to an increased risk of type 2 diabetes because higher consumption levels may increase body weight, the concentrations of certain fats (i.e., triglycerides) in the blood, and blood pressure (Wannamethee and Shaper 2003; Wannamethee et al. 2003).

## Neuropsychiatric Conditions

One of the neuropsychiatric conditions associated with alcohol consumption is epilepsy, which is defined as an enduring predisposition for epileptic seizures and requires the occurrence of at least one seizure for a diagnosis. Alcohol consumption is associated with epilepsy, whereas alcohol withdrawal can cause seizures but not epilepsy (Hillbom et al. 2003).^5^ Observational research has found that a consistent dose-response relationship exists between alcohol consumption and the risk of epilepsy (see figure 3). Multiple possible pathways may underlie this relationship. In particular, alcohol consumption may have a kindling effect, where repeated withdrawals from alcohol consumption by heavy drinkers may lower the threshold for inducing an epileptic episode (Ballenger and Post 1978). Alternatively, heavy alcohol consumption may increase the risk of epilepsy by causing shrinkage of brain tissue (i.e., cerebral atrophy) (Dam et al. 1985), cerebrovascular infarctions, lesions, head traumas, and changes in neurotransmitter systems and ionic balances (Barclay et al. 2008; Dam et al. 1985; Freedland and McMicken 1993; Rathlev et al. 2006).

Another neuropsychiatric disorder considered to be causally linked to alcohol consumption is unipolar depressive disorder. This association is supported by the temporal order of the two conditions, consistency of the findings, reversibility with abstinence, biological plausibility, and the identification of a dose-response relationship. One study determined the risk of depressive disorders to be increased two- to threefold in alcohol-dependent people (see Rehm and colleagues [2003*a*] for an examination of the causal criteria). The alcohol-attributable morbidity and mortality from unipolar depressive disorder currently cannot be calculated because the relationship may be confounded by several factors, including a genetic predisposition, environmental factors (i.e., an underlying disorder or environmental exposure that may contribute to both heavy alcohol use and depressive disorders), and potential self-medication with alcohol by individuals with unipolar depressive disorders (Grant and Pickering 1997; Rehm et al. 2004). Research findings suggest that all of these pathways may play a role. The pathways for the association between alcohol and unipolar depressive disorder in which alcohol does not play a causal role only affect the measurement of the alcohol-based RR for unipolar depressive disorder; however, they do not contradict the notion that alcohol is causally related to the development of unipolar depressive disorder via other pathways. This conclusion results from the observation that depressive symptoms increase markedly during heavy-drinking occasions and disappear or lessen during periods of abstinence (Rehm et al. 2003*a*).

Numerous studies also have examined the association between alcohol and Alzheimer’s disease and vascular dementia.^6^ These analyses generally have determined a beneficial effect of alcohol, which has been attributed to alcohol’s ability to prevent ischemic events in the circulatory system (Peters et al. 2008; Tyas 2001). However, studies of these associations have generated highly heterogeneous results, and the design and statistical analyses of these studies make it impossible to rule out the potential effects of confounding factors (Panza et al. 2008; Peters et al. 2008).

### Cardiovascular and Circulatory Diseases

**Figure 4:**
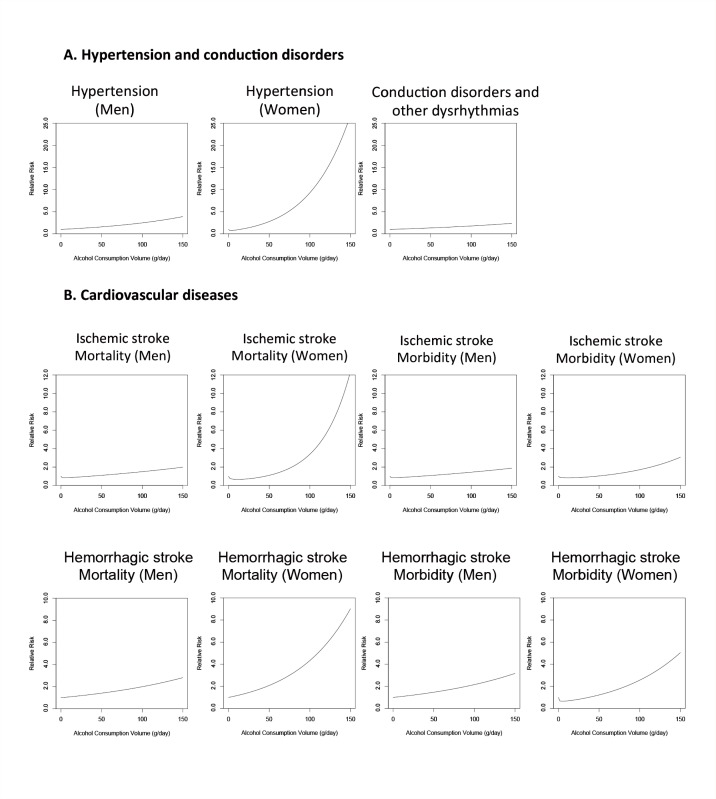
The relationship between increasing amounts of average daily alcohol consumption and the relative risk for cardiovascular diseases (i.e., hypertension, conduction disorders, and ischemic and hemorrhagic stroke), with lifetime abstainers serving as the reference group. For both hypertension and hemorrhagic and ischemic stroke, the relationship differs between men and women. Moreover, for both ischemic and hemorrhagic stroke, the influence of alcohol consumption on mortality is much greater than the influence on morbidity, at least in women. In men, no such difference appears to exist. SOURCE: Lim et al. 2012.

Alcohol consumption affects multiple aspects of the cardiovascular system, with both harmful and protective effects. These include the following (figure 4):

- Increased risk of hypertension (at all consumption levels for men and at higher consumption levels for women);
- Increased risk of disorders that are caused by abnormalities in the generation and disruption of the electrical signals that coordinate the heart beat (i.e., conduction disorders and other dysrhythmias);
- Increased risk of cardiovascular disease, such as stroked caused by blockage of blood vessels in the brain (i.e., ischemic stroke) (at a higher volume of consumption) or rupture of blood vessels (i.e., hemorrhagic stroke); and
- Protective effects (at lower levels of consumption) against hypertension in women and against ischemic heart disease and ischemic stroke in both men and women.

The specific biological pathways through which alcohol consumption interacts with the cardiovascular system are not always clear, but several mechanisms have been identified that may play a role. These include increased blood concentrations of HDLs, effects on cellular signaling, decreased blood clot formation by platelets, and increased blood clot dissolution through enzyme action (Zakhari 1997). More indirect effects also may play a role. For example, alcohol may increase the risk of hypertension by enhancing the activity of the sympathetic nervous system, which results in greater constriction of the blood vessels and makes the heart contract more strongly. In addition, alcohol possibly decreases the sensitivity of the body’s internal blood pressure sensors (i.e., baroreceptors), thereby diminishing its ability to regulate blood pressure.

Alcohol’s protective effects against the risk of ischemic heart disease as well as against hypertension in women is hypothesized to result from its ability to increase HDL levels and/or reduce platelet aggregation on arterial walls. Differences in the effects of alcohol in men and women may stem from differing drinking patterns, with men more likely to engage in binge drinking, even at low average levels of consumption. These heavy-drinking occasions may lead to an increased risk of hypertension for men compared with women at similar alcohol consumption levels (Rehm et al. 2003*b*).

Alcohol’s effect on hypertension also contributes to the risk of hemorrhagic stroke (Taylor et al. 2009), with a hypothesized dose-response effect. The mortality and morbidity from alcohol-attributable hemorrhagic stroke differ by sex (see figure 4). As with hypertension, differences in drinking pattern between men and women most likely are responsible for the differing RR functions for hemorrhagic stroke by sex. Three possible explanations have been put forth to explain the effects of drinking pattern on RR:

- Heavy drinkers also may have other comorbidities that may increase the probability of a fatal hemorrhagic stroke.
- Alcohol consumption may worsen the disease course through biological mechanisms and by decreasing compliance with medication regimens.
- Alcohol’s effects on morbidity may be underestimated because of a stigmatization of heavy alcohol consumption in women, thereby potentially decreasing the probability that female heavy drinkers will be treated for stroke.

Large cohort studies and meta-analyses have shown that alcohol consumption leads to an increase in the risk for conduction disorders and dysrhythmias (Samokhvalov et al. 2010*b*). These effects are caused by changes in the electrical activity of the heart, including direct toxic effects of alcohol on the heart (i.e., cardiotoxicity), excessive activity of the sympathetic nervous system (i.e., hyperadrenergic activity) during drinking and withdrawal, impairment of the parasympathetic nervous system (i.e., of vagal tone), and increase of intra-atrial conduction time (Balbão et al. 2009).

**Figure 5:**
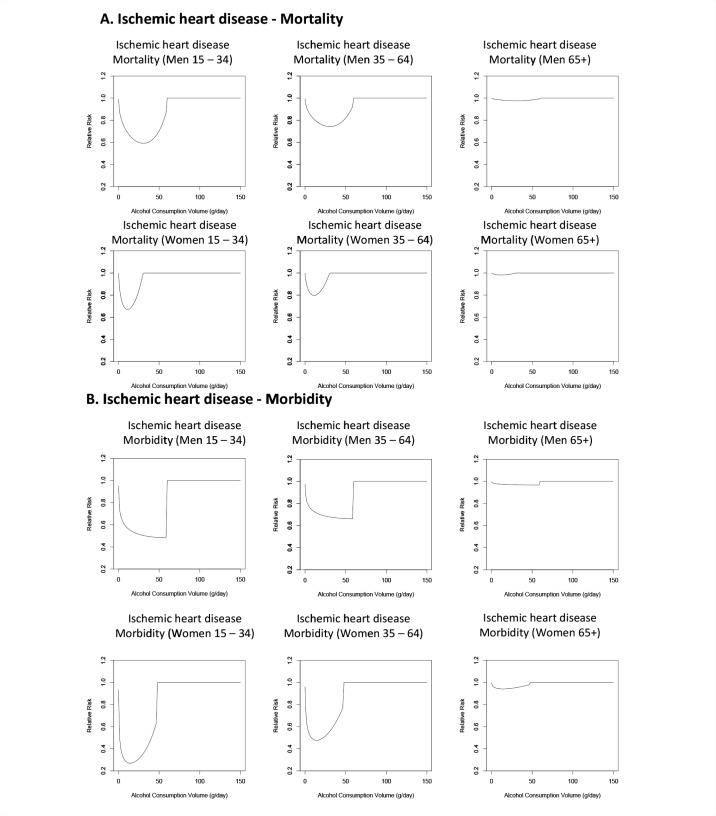
The relationship between increasing amounts of average daily alcohol consumption and the relative risk for ischemic heart disease, with lifetime abstainers serving as the reference group. Low to moderate alcohol consumption has a beneficial effect on both mortality and morbidity from ischemic heart disease. However, the specific effects depend on both the gender and the age of the drinker, with the greatest beneficial effects of low-to-moderate consumption seen on morbidity from ischemic heart disease in women ages 15 to 34. SOURCE: Lim et al. 2012.

Alcohol interacts with the ischemic system to decrease the risk of ischemic stroke and ischemic heart disease at low levels of consumption; however, this protective effect is not observed at higher levels of consumption. As mentioned above, alcohol exerts these effects mainly by increasing levels of HDL, preventing blood clots, and increasing the rate of breakdown of blood clots. However, binge drinking, even by light to moderate drinkers, leads to an increased risk of ischemic events by increasing the probability of clotting and abnormal contractions of the heart chambers (i.e., ventricular fibrillation). As with hemorrhagic stroke, alcohol has different effects on morbidity than on mortality related to ischemic events (see figure 5). Thus, meta-analyses of alcohol consumption and the risk of ischemic heart disease (Roerecke and Rehm 2012) and ischemic stroke (Taylor et al. 2009) found a larger protective effect for morbidity than for mortality related to these conditions. One possible explanation for this observation, in addition to those listed above for hemorrhagic stroke, is that patients in the morbidity studies may be younger at the time of the stroke than those in mortality studies. Despite the increased risk for ischemic heart disease at higher levels of alcohol consumption noted in observational studies (see Roerecke and Rehm 2012 for the most up-to-date meta-analysis), there was not enough evidence for a detrimental effect of alcohol consumption on ischemic heart disease for it to be modeled in the 2005 GBD study.

Moreover, the observational studies investigating the link between alcohol consumption and ischemic events had several methodological flaws, and the RR functions for ischemic events, especially ischemic heart disease, therefore are not well defined. A meta-analysis conducted by Roerecke and Rehm (2012) observed a substantial degree of heterogeneity among all consumption levels, pointing to a possible confounding effect of heavy drinking. In addition, previous observational studies have been limited by the inclusion of “sick quitters” in the reference groups, who have an increased risk of ischemic events compared with lifetime abstainers.

### Digestive Diseases

**Figure 6:**
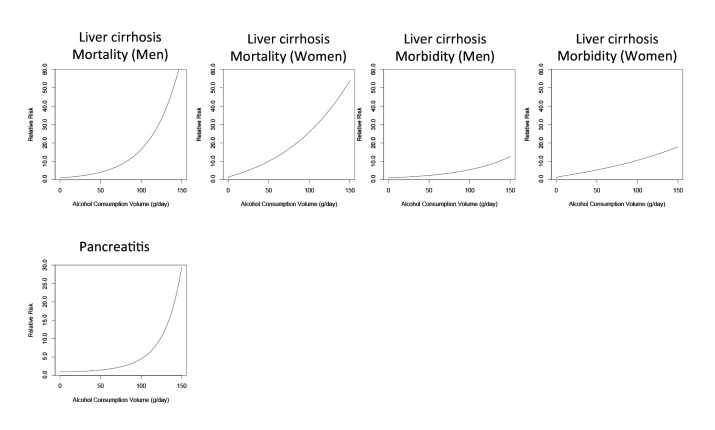
The relationship between increasing amounts of average daily alcohol consumption and the relative risk for digestive diseases (i.e., liver cirrhosis and pancreatitis), with lifetime abstainers serving as the reference group. For liver cirrhosis, alcohol’s effects on mortality are greater than those on morbidity, and slight differences exist between the effects in men and women. SOURCE: Lim et al. 2012.

Alcohol is associated with various liver diseases and is most strongly related to fatty liver, alcoholic hepatitis, and cirrhosis. The association between the risk of liver cirrhosis and alcohol consumption has long been recognized (see figure 6). The main biological mechanism contributing to this liver damage likely involves the breakdown of ethanol in the liver through oxidative and nonoxidative pathways that result in the production of free radicals, acetaldehyde, and fatty acid ethyl esters, which then damage liver cells (Tuma and Casey 2003). Given the same amount of alcohol consumption, alcohol increases the risk of mortality from liver cirrhosis more steeply than the risk of morbidity because it worsens the course of liver disease and has a detrimental effect on the immune system (Rehm et al. 2010*c*).

Alcohol consumption also has been linked to an increase in the risk for acute and chronic pancreatitis. Specifically, heavy alcohol consumption (i.e., more than, on average, 48 grams pure ethanol, or about two standard drinks, per day) leads to a noticeably elevated risk of pancreatitis, whereas consumption below 48 grams per day is associated with a small increase in risk of pancreatitis (see figure 6). Higher levels of alcohol consumption may affect the risk of pancreatitis through the same pathways that cause liver damage, namely the formation of free radicals, acetaldehyde, and fatty acid ethyl esters during the metabolism of alcohol in damaged pancreatic acinar cells (Vonlaufen et al. 2007).

### Psoriasis

Psoriasis is a chronic inflammatory skin disease caused by the body’s own immune system attacking certain cells in the body (i.e., an autoimmune reaction). Although there is insufficient biological evidence to indicate that alcohol is causally linked with psoriasis, many observational studies have determined a detrimental impact of drinking on psoriasis, especially in male patients. Alcohol is hypothesized to induce immune dysfunction that results in relative immunosuppression. In addition, alcohol may increase the production of inflammatory cytokines and cell cycle activators, such as cyclin D1 and keratinocyte growth factor, that could lead to excessive multiplication of skin cells (i.e., epidermal hyperproliferation). Finally, alcohol may exacerbate disease progression by interfering with compliance with treatment regimens (Gupta et al. 1993; Zaghloul and Goodfield 2004).

### Alcohol’s Effects on Other Medication Regimens

Alcohol can affect cognitive capacity, leading to impaired judgment and a decreasing ability to remember important information, including when to take medications for other conditions (Braithwaite et al. 2008; Hendershot et al. 2009; Parsons et al. 2008). Although the relationship between alcohol consumption and adherence to treatment regimens mainly has been studied in regards to adherence to HIV antiretroviral treatment (Braithwaite and Bryant 2010; Hendershot et al. 2009; Neuman et al. 2012), research also has shown that alcohol consumption and alcohol misuse impact adherence to medications for other chronic diseases, with significant or almost-significant effects (Bates et al. 2010; Bryson et al. 2008; Coldham et al. 2002; Verdoux et al. 2000). Thus, for diseases or conditions managed by pharmacotherapy, alcohol consumption likely is associated with increased morbidity and even mortality (if nonadherence to the medication could be fatal) if drinking results in nonadherence to medication regimens.

### Impact of Sex, Race, and Age on the Association of Alcohol Consumption with Chronic Diseases

Given the same amount of alcohol consumed, men and women can have differing morbidity and mortality from alcohol-related chronic disease and conditions. These differences may be related to the pharmacokinetics of alcohol in men and women. Women generally have a lower body water content than men with the same body weight, causing women to reach higher blood alcohol concentrations than men after drinking an equivalent amount of alcohol (Frezza et al. 1990; Taylor et al. 1996). Moreover, women appear to eliminate alcohol from the blood faster than do men, possibly because they have a higher liver volume per unit body mass (Kwo et al. 1998; Lieber 2000). In addition to these pharmacokinetic factors, hormonal differences also may play a role because at least in the case of liver disease, alcohol-attributable harm is modified by estrogen. However, hormonal influences on alcohol-related risks are not yet fully understood (Eagon 2010).

As noted previously, a deficiency of the ALDH2 enzyme in people carrying the *ALDH2 Lys487* allele contributes to an elevated risk of cancer from alcohol consumption. Because alcohol metabolism also plays a role in many other chronic diseases, the *ALDH2 Lys487* allele also may increase the risk for digestive diseases. The heterogeneity of risk caused by this allele, which is more prevalent in Asian populations, may lead to incorrect measurements of the risk for cancer and digestive disease outcomes in countries with a small Asian population, and will lead to incorrect results if the RRs from these countries are applied to Asian populations (Lewis and Smith 2005; Oze et al. 2011).

Because the pathology of alcohol-related ischemic heart disease is affected by the age of the drinker (Lazebnik et al. 2011), differences also may exist in the risk of ischemic heart disease in different age groups. Preliminary research assessing this issue across multiple studies has found that the association between alcohol consumption and the resulting risk for ischemic heart disease does indeed differ by age (see figure 5). However, no meta-analyses to date have investigated the effects of alcohol consumption on the risk of morbidity and mortality in different age groups for other chronic diseases and conditions. Accordingly, research is needed to assess if the varying relationship between alcohol consumption and ischemic heart disease in different age groups results from biological differences in pathology or from differences in drinking patterns. Additionally, research is needed to assess if age modifies the risk relationships between alcohol and other diseases.

## Estimating the Alcohol-Attributable Fractions of Chronic Diseases and Conditions

When assessing the risk of chronic diseases and conditions that are related to alcohol consumption in some, but not all, cases, one of the variables frequently analyzed is the alcohol-attributable fraction (AAF)—that is, the proportion of cases that can be attributed to the patient’s alcohol consumption. Because alcohol consumption can be modeled using a continuous distribution (Kehoe et al. 2012; Rehm et al. 2010*b*), the calculation of the alcohol-attributable burden of disease uses a continuous RR function.^7^ Thus, the AAFs for chronic diseases and conditions can be calculated using the following formula:

$$\mathrm{AAF}=\frac{P_{\mathrm{abs}}+P_{\mathrm{form}}RR_{\mathrm{form}}+\int_{+0}^{\max}P(x)\;RR(x)\;dx-1}{P_{\mathrm{abs}}+P_{\mathrm{form}}RR_{\mathrm{form}}+\int_{+0}^{\max}P(x)\;RR(x)\;dx}$$

In this formula, *P_abs_* represents the prevalence of the disease among lifetime abstainers, *P_form_* is the prevalence among former drinkers, *RR_form_* is the relative risk for former drinkers, P(*x*) is the prevalence among current drinkers with an average daily alcohol consumption of *x*, and *RR*(*x*) is the relative risk for current drinkers with an average daily alcohol consumption of x. These AAFs vary greatly depending on alcohol exposure levels. (For examples of AAFs and information on the calculation of the 95 percent confidence intervals for chronic diseases and conditions see Gmel and colleagues [2011]).

## Limitations of RR Functions for Chronic Diseases and Conditions

The chronic disease RR functions outlined in figures 2 to 6 are derived from the most up-to-date and rigorous meta-analyses in which the risk of a disease (i.e., for mortality and, where possible, morbidity) was provided by alcohol consumption as a continuous function (for more details on the meta-analysis methods used in each study, see the original articles cited in table 2). However, the RR functions and the relationship between alcohol consumption and the risk of chronic diseases and conditions are biased by multiple factors. First, the RRs can be limited by poor measurement of alcohol exposure, outcomes, and confounders. Research on alcohol consumption patterns and disease is scarce, and only few studies have investigated the effects of drinking patterns on chronic diseases and conditions. Thus, the chronic disease and condition RRs presented in this article may be confounded by drinking patterns, which are correlated to overall volume of alcohol consumption. Additionally, the volume of alcohol consumed generally is poorly measured, with many medical epidemiology studies measuring alcohol consumption only at baseline. As a result, these analyses do not include measures of the volume of alcohol consumed during the medically relevant time period, which may encompass several years. For example, in the case of cancer, the cumulative effects of alcohol may take many years before an outcome is observed. Likewise, many of the larger cohort studies only use single-item, semi-quantitative food questionnaires that measure either frequency or volume of consumption.

Second, medical epidemiology studies typically suffer from poorly defined reference groups (Rehm et al. 2008). Thus, such studies typically only measure alcohol consumption at one point or during one time period in a participant’s life and classify, for example, all people who do not consume alcohol during the reference period as abstainers, even though it is essential to separate ex-drinkers, lifetime abstainers, and very light drinkers. As a result, these measurements of alcohol consumption may lead to incorrect risk estimates because the groups of nondrinkers in these studies have heterogeneous risks for diseases (Shaper and Wannamethee 1998). The potential significance of this issue is underscored by previous research indicating that more than 50 percent of those participants who identified themselves as lifetime abstainers in medical epidemiology studies also had reported lifetime drinking in previous surveys (Rehm et al. 2008).

Third, chronic disease and condition outcomes in medical epidemiology studies also frequently are poorly measured, most often by means of self-reporting. Additionally, other confounding factors, such as relevant, non-substance use–related confounders, often are not controlled for.

Fourth, RR estimates for chronic diseases and conditions resulting from alcohol consumption frequently are hampered by weak study designs that base estimates of alcohol-related risks on nonexperimental designs (i.e., case-control and cohort studies). These study designs are limited by factors that cannot be controlled for and which may lead to incorrect results. For example, experimental studies on the effects of antioxidants have failed to confirm the protective effects of such agents found in observational studies (Bjelakovic et al. 2008). Furthermore, the sampling methodology of many of the cohort studies that were used in the meta-analyses for the above-presented RRs is problematic, especially when studying the effects of alcohol consumption. Many of the cohorts in these studies were from high-income countries and were chosen based on maximizing follow-up rates. Although the chosen cohorts exhibited variation in average daily alcohol consumption, little variation was observed in drinking patterns and other potential moderating lifestyle factors.

The overall effect of these limitations on the RRs and AAFs, and on the estimated burden of mortality and morbidity calculated using these RRs, currently is unclear. In order to investigate the effect of these biases, studies should be undertaken that combine better exposure measures of alcohol consumption with state-of-the-art outcome measures in countries at all levels of economic development. These studies are important, not only for understanding the etiology of alcohol-related chronic diseases and conditions, but also for formulating prevention measures (Stockwell et al. 1997).

## Limitations of AAFs for Chronic Diseases and Conditions

In most studies assessing AAFs for chronic diseases and conditions, the AAF for an outcome is calculated as if the health consequences of alcohol consumption are immediate. Indeed, for most chronic diseases and conditions, including even diseases such as cirrhosis, a large degree of the effects caused by changes in alcohol consumption can be seen immediately at the population level (Holmes et al. 2011; Leon et al. 1997; Zatonski et al. 2010; for a general discussion see Norström and Skog 2001; Skog 1988). For cancer, however, the situation is different. The effects of alcohol consumption on the risk of cancer only can be seen after years, and often as long as two decades. Nevertheless, for the purpose of illustrating the entire alcohol-attributable burden of disease it is important to include cancer deaths, because they account for a substantial burden. For example, a recent large study found that in Europe 1 in 10 cancers in men and 1 in 33 cancers in women were alcohol related (Schütze et al. 2011). Therefore, in the interpretation of alcohol’s effect on mortality and burden of disease in this article, the assumption that there has been uniform exposure to alcohol for at least the previous two decades must be kept in mind.

Another limitation to calculating the burden of chronic diseases and conditions attributable to alcohol consumption is the use of mainly unadjusted RRs to determine the AAFs. The RR formulas were developed for risks and were adjusted only for age (see Flegal et al. 2006; Korn and Graubard 1999; Rockhill and Newman 1998), although many other socio-demographic factors are linked with both alcohol consumption and alcohol-related harms (see figure 1). However, two arguments can be made to justify the use of mainly unadjusted RR formulas in the 2005 GBD study. First, in risk analysis studies (Ezzati et al. 2004) almost all of the underlying studies of the different risk factors only report unadjusted risks. Relying on adjusted risks would severely bias the estimated risk functions because only a small proportion of generally older studies could be included. Second, most of the analyses of alcohol and the risk of chronic diseases and conditions show no marked differences after adjustment (see Rehm et al. 2010*b*). However, the need for adjustment to the RRs may change when other dimensions of alcohol consumption, such as irregular heavy-drinking occasions, are considered with respect to ischemic heart disease.

## Conclusions

There are limitations to the current ability to estimate the burden of chronic diseases and conditions attributable to alcohol consumption. The comparative risk assessment study within the GBD study only can determine this burden based on current knowledge of alcohol consumption and risk and mortality patterns at a global level. More detailed, country-specific estimates often are limited by the validity of the available consumption and mortality data. As more studies are published, it is likely that new confounders will be discovered for some of the relationships between alcohol consumption and various chronic diseases and conditions. The results from such new studies then may be used in meta-analyses of the effect of alcohol in diseases where alcohol only plays a small role, such as bladder, endometrial, and ovarian cancer. New studies also may lead to the recognition of a causal link between alcohol consumption and other diseases. Furthermore, new confounders and new studies may disprove the relationship between alcohol consumption and certain diseases that currently are considered to be causally linked.

Although there are limitations to the current methodology used to estimate the alcohol-attributable burden of chronic diseases and conditions, the limitations discussed in this article do not affect the overall conclusion that alcohol consumption is related to a considerable number of chronic diseases and conditions and contributes to a substantial amount of the global burden of chronic diseases and conditions. Therefore, alcohol consumption should be considered in developing intervention strategies aimed at reducing the burden of chronic diseases and conditions.

## Figures and Tables

**Table 1:** Chronic Diseases and Conditions That Are, by Definition, Alcohol Attributable (i.e., Require Alcohol Consumption As a Necessary Cause)

**ICD–10 Code**	**Disease**
F10	Mental and behavioral disorders attributed to the use of alcohol
F10.0	Acute intoxication
F10.1	Harmful use
F10.2	Dependence syndrome
F10.3	Withdrawal state
F10.4	Withdrawal state with delirium
F10.5	Psychotic disorder
F10.6	Amnesic syndrome
F10.7	Residual and late-onset psychotic disorder
F10.8	Other mental and behavioral disorders
F10.9	Unspecified mental and behavioral disorder
G31.2	Degeneration of nervous system attributed to alcohol
G62.1	Alcoholic polyneuropathy
G72.1	Alcoholic myopathy
I42.6	Alcoholic cardiomyopathy
K29.2	Alcoholic gastritis
K70	Alcoholic liver disease
K70.0	Alcoholic fatty liver
K70.1	Alcoholic hepatitis
K70.2	Alcoholic fibrosis and sclerosis of liver
K70.3	Alcoholic cirrhosis of liver
K70.4	Alcoholic hepatic failure
K70.9	Alcoholic liver disease, unspecified
K85.2	Alcohol-induced acute pancreatitis
K86.0	Alcohol-induced chronic pancreatitis
P04.3	Fetus and newborn affected by maternal use of alcohol
Q86.0	Fetal alcohol syndrome (dysmorphic)

**Table 2:** Chronic Diseases and Conditions for Which Alcohol Consumption Is a Component Cause, Identified by Various Meta-Analyses and Reviews and Listed in the 2005 Global Burden of Disease (GBD) Study

**No. of 2005 GBD Code**	**Disease**	**ICD–10**	**Effect**	**Level of Evidence Meta-Analysis**	**Used if Included in the GBD Study**
**IIA**	**Malignant neoplasms**				
IIA1	Mouth cancer	C00–C08	Detrimental	Causally related	International Agency for Research on Cancer 2008 (based on relative risks from Corrao et al. 2004)
IIA2	Nasopharynx cancer and other pharynx cancers	C09–C13	Detrimental	Causally related	International Agency for Research on Cancer 2008 (based on relative risks from Corrao et al. 2004)
IIA3	Esophagus cancer	C15	Detrimental	Causally related	International Agency for Research on Cancer 2008 (based on relative risks from Corrao et al. 2004)
IIA4	Stomach cancer	C16	Detrimental	Insufficient causal evidence	
IIA5	Colon and rectum cancers	C18–C21	Detrimental	Causally related	International Agency for Research on Cancer 2008 (based on relative risks from Corrao et al. 2004)
IIA6	Liver cancer	C22	Detrimental	Causally related	International Agency for Research on Cancer 2008 (based on relative risks from Corrao et al. 2004)
IIA9	Larynx cancer	C32	Detrimental	Causally related	International Agency for Research on Cancer 2008 (based on relative risks from Corrao et al. 2004)
IIA10	Trachea, bronchus, and lung cancers	C33–C34	Detrimental	Insufficient causal evidence	
IIA13	Breast cancer (women only)	C50	Detrimental	Causally related	International Agency for Research on Cancer 2008 (based on relative risks from Corrao et al. 2004)
IIA16	Ovarian cancer	C56	Detrimental	Insufficient causal evidence	
IIA17	Prostate cancer	C61	Detrimental	Insufficient causal evidence	
IIA19	Kidney and other urinary organ cancers	C64–C66, C68 (except C68.9)	Beneficial (renal cell carcinoma only)	Insufficient causal evidence	
IIA23	Hodgkins lymphoma	C81	Beneficial	Insufficient causal evidence	
IIA24	Non-Hodgkins lymphoma	C82–C85, C96	Beneficial	Insufficient causal evidence	
**IIB**	**Other neoplasms**	D00–D48 (except D09.9, D37.9, D38.6, D39.9, D40.9, D41.9, 48.9)	Detrimental		
**IIC**	**Diabetes**	E10–E13	Beneficial (however, this depends on drinking patterns and volume of consumption)	Causally related	(Baliunas et al. 2009)
**IIE**	**Mental and behavioral disorders**				
IIE1	Unipolar depressive disorders	F32–F33, F34.1	Detrimental	Causally related	
**IIF**	**Neurological conditions**				
IIF1	Alzheimer’s disease and other dementias	F01–F03, G30–G31	Conflicting evidence (mainly beneficial)	Insufficient causal evidence	
IIF3	Epilepsy	G40–G41	Detrimental	Causally related	(Samokhvalov et al. 2010a)
**IIH**	**Cardiovascular and circulatory diseases**				
IIH2	Hypertensive heart disease	I11–I13	Detrimental (however, this depends on drinking patterns and volume of consumption)	Causally related	(Taylor et al. 2010)
IIH3	Ischemic heart disease	I20–I25	Beneficial (however, this depends on drinking patterns and volume of consumption)	Causally related	(Roerecke and Rehm 2010)
**IIH4**	**Cerebrovascular diseases**				
IIH4a	Ischemic stroke	I63–I67, I69.3	Beneficial (however, this depends on drinking patterns and volume of consumption)	Causally related	(Patra et al. 2010)

## Footnotes

1 Ischemic cardiovascular diseases are those caused by a blockage of blood vessels, resulting in a loss of blood supply to the tissue serviced by the affected blood vessels.

2 The GBD Study is a project that aims to provide a consistent and comparative description of the global burden of diseases and injuries and the risk factors that cause them.

3 There are two main types of diabetes. Type 1 diabetes results from the body’s failure to produce insulin, and patients therefore regularly must inject insulin. This type also is known as juvenile diabetes because of its early onset, or insulin-independent diabetes. Type 2 diabetes results from insulin resistance, which develops when the cells fail to respond properly to insulin. It develops with age and therefore also is referred to as adult-onset diabetes.

5 Seizures are excluded from the 2005 GBD study definition of epilepsy.

6 Vascular dementia, the second most common form of dementia after Alzheimer’s disease, is caused by problems in the supply of blood to the brain. Its most common symptoms include problems with thinking, concentration, and communication; depression and anxiety; physical weakness or paralysis; memory problems; seizures; and periods of severe confusion.

7 The exception to this approach is tuberculosis because only data on categorical alcohol exposure risks are available.
