# Supplementary material for: Chronic Diseases and Conditions Related to Alcohol Use
Source: Alcohol Res. 2014;35(2):155–71. doi: 10.35946/arcr.v35.2.06 (PMC3908707; doi:10.35946/arcr.v35.2.06)
Supplement: Related texts

# Sub article: Economic Costs of Excessive Alcohol Use

**Published:** 2014

This brief sidebar summarizes recent findings on the economic costs of excessive alcohol use and highlights the primary methods used to estimate these costs. The total economic cost of excessive drinking in 2006,^1^ including costs for health care, productivity losses, and costs such as property damage and alcohol-related crime, was estimated to be $223.5 billion (see table) (Bouchery et al. 2011). Prior to this estimate, the last comprehensive analysis reported that the estimated economic costs of excessive drinking were $148 billion in 1992 (Harwood et al. 1998). Data from that report were used to project a cost estimate of $185 billion for 1998^2^ (Harwood 2000). For a review of the global burden of alcohol use, see Rehm and colleagues (2009). For a broader examination of both the methods used to reach the current estimates and details on each of the estimated costs, as well as analysis of the significance and limitations of the study, see Bouchery and colleagues (2011, 2013).

Researchers used updated data, new data sources, and new measurement tools to develop the 2006 estimate. For example, the Alcohol-Related Disease Impact (ARDI) software,^3^ created by the Centers for Disease Control and Prevention, was used to assess the relationship between excessive drinking and various health and social outcomes. This software, originally released in 2004, was designed to allow researchers to calculate alcohol-attributable deaths, years of potential life lost, direct health care costs, indirect morbidity and mortality costs, and non–health-sector costs associated with alcohol misuse.

## How the Costs of Excessive Alcohol Use Are Estimated

As in the 1992 and 1998 reports of estimated costs, the most recent report was based on the “cost-of-illness” approach. That is, costs for health care, productivity losses, and other expenses for 2006 were obtained from national databases, and alcohol-attributable fractions (AAFs) were applied to assess the proportion of costs that could be attributed to excessive alcohol consumption.^4^ For example, researchers would consult a national health care database to determine the cost of liver cirrhosis and multiply this cost by a reasonable AAF to determine the proportion of the total cost that can be attributed to excessive alcohol use. Likewise, costs of lost productivity, crime, and other consequences of excessive alcohol use are estimated based on information gathered from various national surveys and databases.^5^

### Defining Excessive Alcohol Consumption

The recent report estimated the costs of excessive alcohol consumption, which includes binge drinking (four or more drinks per occasion for a woman and five or more drinks per occasion for a man), heavy drinking (more than one drink per day on average for a woman and more than two drinks per day on average for a man), any alcohol consumption by persons younger than 21, and any alcohol consumption by pregnant women. The surveys used generally ask respondents about the 30 days prior to the survey. The focus on excessive drinking, rather than alcohol use disorders, allows for a more comprehensive look at the health and social consequences that are associated with harmful drinking patterns, including a wide range of acute and chronic health problems, productivity losses due to absenteeism, and crimes committed while intoxicated. In some cases, a history of alcohol abuse or dependence was used as a specific indicator of excessive drinking (e.g., productivity losses based on lost earnings).

### Alcohol-Attributable Fractions

AAFs, or the proportion of a condition or outcome that is attributed to excessive alcohol consumption, were used to estimate both health-related costs, including deaths and health care expenditures related to excessive drinking, and the costs of specific criminal offenses.

#### Crime-Specific AAFs

Certain alcohol-related crimes (e.g., driving under the influence of alcohol, public drunkenness, and liquor law violations) are fully attributed to alcohol. For other offenses, researchers estimated the proportion attributable to alcohol based on the percentage of offenders intoxicated at the time of their offense (according to self-reported alcohol-consumption data from surveys of inmates). For homicide, researchers used the AAF from ARDI because it takes into account drinking by both the perpetrator and the victim.

## The Costs of Excessive Alcohol Use

Of the total estimated cost of excessive alcohol use for 2006, lost productivity represents 72.2 percent,^6^ health care costs represent 11.0 percent, criminal justice system costs make up 9.4 percent, and other consequences make up 7.5 percent (see figure).

## Figures and Tables

**Table:** 

**Table:** Total Economic Costs of Excessive Alcohol Consumption in the United States, 2006

**Cost Category**	**Total Cost**
**Health Care Costs**	
Alcohol Abuse and Dependence	$10,668.457
Primary Diagnoses Attributable to Alcohol	8,526.822
Inpatient Hospital	5,115.568
Physician Office and Hospital Ambulatory Care	1,195.946
Nursing Home Care	1,002.888
Retail Pharmacy and Other Health Professional	1,212.420
Fetal Alcohol Syndrome	2,538.004
Other Health System Costs	2,822.308
Prevention and Research	1,207.120
Training	29.527
Health Insurance Administration	1,585.660
**Total Health Care Costs**	**$24,555.591**
**Productivity Losses**	
Impaired Productivity	$83,695.036
Traditional Earnings	74,101.827
Household Productivity	5,355.629
Absenteeism	4,237.580
Institutionalization/Hospitalization	2,053.308
Mortality	65,062.211
Incarcerations	6,328.915
Victims of Crime	2,092.886
Fetal Alcohol Syndrome	2,053.748
**Total Productivity Losses**	**$161,286.103**
**Other Effects on Society**	
Crime Victim Property Damage	$439.766
Criminal Justice System	20,972.690
Motor Vehicle Crashes	13,718.406
Fire Losses	2,137.300
FAS Special Education	368.768
**Total Other Effects**	**$37,636.930**

**Total**	**$223,478.624**

## Footnotes

1 The most recent year for which data were available.

2 The estimates given for 1992 and 1998 have not been adjusted for inflation.

3 The software is available online at: http://apps.nccd.cdc.gov/DACH_ARDI/Default/Default.aspx.

4 Although cost-of-illness studies remain a popular tool for estimating the impact of alcohol abuse, researchers have criticized such studies as an invalid measure of alcohol’s impact on society. For a recent review of arguments against this approach, see Mäkelä (2012).

5 For information on the main data sources used in the report, see Bouchery and colleagues (2011, 2013).

6 Although the methods used to estimate productivity losses attributed to premature mortality are consistent with previous cost-of-illness studies, alternative methods with greater support from economists (i.e., the so-called willingness-to-pay approach) would yield much larger cost estimates.

## References

[b110-arcr-35-2-155] Bouchery E, Harwood H, Sacks J (2011). Economic costs of excessive alcohol consumption in the U.S., 2006. American Journal of Preventive Medicine.

[b111-arcr-35-2-155] Bouchery E, Simon C, Harwood H Economic Costs of Excessive Alcohol Consumption in the United States, 2006.

[b112-arcr-35-2-155] Harwood H (2000). Updating Estimates of the Economic Costs of Alcohol Abuse in the US: Estimates, Update Methods and Data.

[b113-arcr-35-2-155] Harwood H, Fountain D, Livermore G The Economic Costs of Alcohol and Drug Abuse in the U.S., 1992.

[b114-arcr-35-2-155] Mäkelä K (2012). Cost-of-alcohol studies as a research programme. Nordic Studies on Alcohol and Drugs.

[b115-arcr-35-2-155] Rehm J, Mathers C, Popova S (2009). Global burden of disease and injury and economic cost attributable to alcohol use and alcohol-use disorders. Lancet.
